# The Effectiveness of Quadratus Lumborum and Fascia Iliaca Blocks on Patient Outcomes in Hip Arthroplasty

**DOI:** 10.1155/2024/4518587

**Published:** 2024-06-19

**Authors:** Cameron Gauhl, Seaneen McDougall

**Affiliations:** ^1^School of Medicine, University of Dundee, Dundee DD1 4HN, UK; ^2^School of Science and Engineering, University of Dundee, Dundee DD1 4HN, UK

## Abstract

Hip arthroplasty is a common procedure with high costs and difficult rehabilitation. It causes postoperative pain, and this can reduce mobility which extends in-patient time. An optimal analgesia regime is crucial to identify. Opioids produce effective pain relief but are associated with nausea, vomiting, and respiratory depression which can hinder physiotherapy and discharge. Finding alternatives has been of interest in recent years, particularly fascial blocks. These are anaesthetic injections beneath fascia which spread to nerves providing pain relief from surgery and are used with a general or spinal anaesthetic. Two of these blocks which are of interest to total hip arthroplasty are the quadratus lumborum block and fascia iliaca block. Studies have investigated the effectiveness of these blocks through patient factors, primarily pain scores, opioid consumption, and other secondary outcomes such as ambulation and length of stay. This review takes a narrative approach and investigates the literature around the topic. Pain and opioid consumption were the most widely reported outcomes, reported in 90% and 86% of studies. 83% of these studies reported positive effects on pain scores when FIB was utilised. 80% of these studies reported positive effects on opioid consumption when FIB was used. When QLB block was utilised, pain and opioid consumption were positively impacted in 82% of studies. This paper has been written with the intention of reviewing current literature to give an impression of the effectiveness of the blocks and propose potential areas for future work on the blocks.

## 1. Introduction

As the number of individuals living to an older age increases, so does the need for disease treatment and surgery intervention designed to sustain and improve quality of life [1, 2]. Finding the most optimal way to treat diseases associated with aging is crucial [2, 3]. Hip replacement surgery, also known as hip arthroplasty, is one procedure that is becoming ever more common in the elderly. In Scotland, the incidence of hip replacement surgery is believed to have increased by more than double since 2001 [2]. Postoperative pain is particularly difficult to manage due to the complex nerve supply of the hip joint, surrounding muscles, and cutaneous innervation. This leads to difficult rehabilitation for patients, heavy pain killers, and often poor satisfaction following surgery [4]. Opioids are often used in the management of pain associated with hip replacement surgery both before and after surgery [4, 5]. Despite these opioids being reasonably effective, they do come with their own set of complications such as respiratory depression, nausea, vomiting, confusion, and drowsiness [4, 5], as well as the potential for patient dependence. These side effects may be manageable and bearable for some with minimal hindrance on their rehabilitation. However, many patients struggle with mobilisation and postoperative physiotherapy due to the side effects of these medications or ineffective pain relief [6]. Since early mobilisation after hip arthroplasty is encouraged and seen as a good prognostic factor [6], finding a pain medication regime which promotes and enables this is likely to be extremely beneficial to patients undergoing this procedure, allowing the impact on their daily living activities to be minimised [4]. A technique which is continuing to show promise in this area is fascial anaesthetic blocks [7, 8]. These are used in conjunction with primary anaesthesia such as a spinal anaesthetic or general anaesthetic and involve injecting analgesia within a fascial sheath which allows spread of the anaesthetic and blocking of nerve structures within the region [9, 10]. Two fascial block techniques which have been investigated for total hip arthroplasty (THA) are the quadratus lumborum block (QLB) and the fascia iliaca block (FIB) [6–9]. These techniques are similar in that they both involve anaesthetic injections targeting the lumbar region nerve structures [9, 10]. However, due to the relevant surrounding anatomy, the QLB has been found to target the higher nerve roots such as L1/2 but with variable spread [11], with FIB usually targeting lower nerves of the plexus such as the femoral, obturator, and lateral cutaneous nerves [7]. They have both shown to improve postoperative pain, but their optimal usage is yet to be determined [8, 9]. As fascial blocks have shown promise, it would be advantageous to identify a standardised approach to their use in order to improve patient pain, mobilisation, opioid consumption, and overall satisfaction after THA [4, 5]. This review investigates the literature with a focus on patient outcomes and whether the use of a fascial block positively or negatively impacts factors such as pain scores, opioid consumption, and ambulation. This article aims to review the current literature and provide a current view of the effectiveness of the QLB and FIB for THA. It also identifies gaps in the current understanding, together with providing suggestions for future work to address these gaps.

## 2. Materials and Methods

To conduct this literature review, search terms were formulated based on background reading of the topic and the aim of the research. Inclusion and exclusion criteria (Table 1) were formed to ensure the reviewed literature was up to date and relevant. The searches were conducted in February 2023 using Web of Science (Medline), PubMed, and Google Scholar databases.

The search terms were chosen with an aim of obtaining relevant results; therefore, “fascia iliaca” and “quadratus lumborum” made the basis of the searches. They were both truncated to incorporate increased results as varying terms were seen during background reading such as “fascia iliaca block” or “fascia iliaca compartment block.” As the focus is around total hip arthroplasty and how the fascial blocks relate to patient outcomes, the terms “total hip arthroplasty” and “patient outcomes” were added to each of the searches separately. “NOT c^*∗*^ sarean section” was added to the “quadratus lumborum AND patient outcomes” term as it is widely used in the procedure and this removed many irrelevant results.

English language and past 5 years' filters were applied. Papers which were deemed irrelevant based on an initial screen of the title alone were removed. This included titles with “paediatrics,” “knee arthroplasty,” or “abdominal surgery” as these were not of interest to this review. This gave 336 results from both databases combined. The terms were also put through Google Scholar to collate further relevant articles and to widen the scope of articles reviewed. The searches produced thousands of results; therefore, the first 200 results were screened only for each individual search term.

**Figure 1:**
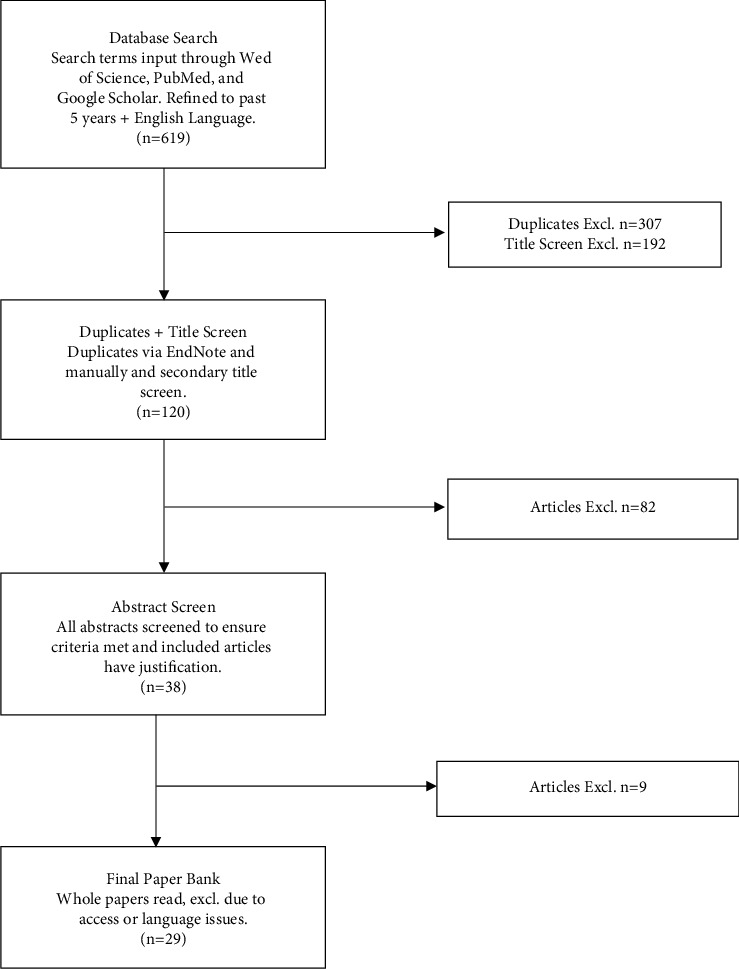
Flow diagram of study selection process. The diagram depicts the stages of study selection described in the methods' section.

This produced 283 papers. All 619 results were collated in EndNote Online referencing tool where duplicates were removed to give 312. From this paper bank, a thorough screen of the titles was again conducted to ensure the papers were relevant. Papers which focused solely on a different analgesia type such as pericapsular nerve group block were removed, as well as papers discussing other surgeries or young patients which were missed in the first screen. Cadaveric studies or anatomical reports were also removed from the analysis as the focus was to investigate patient outcomes. Whilst cadaveric studies have their advantages, they were not relevant to patient outcomes. It was also decided that any papers describing “hip arthroscopy,” “hip fractures,” “femur fractures,” and other techniques, or that did not specify hip arthroplasty were removed as to ensure specificity of the papers reviewed. This reduced the bank to 120. The abstracts were then screened again applying the inclusion/exclusion criteria in Table 1. Criteria were applied for each article to ensure justification for its inclusion was apparent. For example, papers reviewing multiple different analgesia modalities were removed, articles including surgeries such as femur nail/plates and fascial blocks vs. other modalities were also excluded. Furthermore, pharmacokinetics of fascial blocks and drug volume studies were also removed. The number of eligible articles following this stage was 38. The next stage involved reviewing the full articles. Papers were removed due to being revision arthroplasty surgeries, investigating postoperative delirium, study protocols, and 2 articles which could only be located in Chinese. The final paper bank included 29 eligible papers. A flow diagram shows this process in a simplified form (Figure 1).

From these papers, data surrounding the reported patient outcomes were analysed to determine an overall positive, negative, or neutral view of the utilisation of the fascial block. A positive impact would be a reduced pain score or less opioid consumption. For example, if the FIB was used compared to a control, opioid consumption was reduced significantly; this was noted as positive. If pain score was not significantly reduced or increased with the use of a block, this would be negative, and finally if ambulation was improved, however, not clinically significantly, this would be neutral. These are three examples and this was completed for the various patient outcomes reported within papers. These recordings formed the results and platform for discussion.

### 2.1. Block Technique

Varying techniques were utilised throughout the literature for the QLB and FIB blocks. To give the reader an idea of the technique, a brief description of one technique has been included for each block. For the QLB, the patient is laid in the lateral decubitus position, with their nonoperative hip facing upwards [10]. The ultrasound probe is placed transversely to the lateral abdomen in the anterior axillary line and above the iliac crest. The quadratus lumborum, psoas major, and erector spinae muscles must be identified and then the needle is inserted through the quadratus lumborum muscle and into the fascial plane where the anaesthetic is administered [10, 11]. Where the needle is placed and the injection given can vary in this procedure—through the muscle, lateral to the muscle or posterior to the muscle were all noted within the literature [2, 6, 10]. For the FIB, it can be completed superior or inferior to the inguinal ligament, with suprainguinal FIB being undertaken more often. The ultrasound probe is placed along the inguinal sulcus at the level of the femoral vessels [9]. When the femoral nerve is identified, the probe is rotated so it is in the sagittal plane and then it is moved cranially until it is superior to the inguinal ligament [9]. This is completed along the plane of the iliopsoas muscle. The needle is advanced in a cranial direction, placement is confirmed, and then anaesthesia is injected [9]. The FIB is performed with the patient in the supine position [12].

## 3. Results

From the 29 papers reviewed, the most widely reported patient outcomes were pain scores, which reported 90% of the time and opioid consumption and 86% of the time. Of these articles, 89% indicated positive effects on pain scores when using the fascial blocks in THA and 11% were neutral. Positive effects on opioid consumption were reported in 88% of articles, while 12% were neutral. Within the 29 papers, 14 were focused on FIB vs. control, 11 investigated QLB vs. control, and 4 compared FIB to QLB. An overall representation of the positive/negative effects on the main outcomes reported can be seen in Table 2 for each paper included in the review.

**Figure 2:**
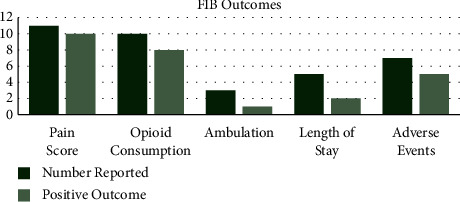
FIB outcomes. The chart displays the number of times an outcome was reported throughout the studies reviewed. The number of times a positive effect on the outcome was reported is plotted beside this to visualise the effects of the fascia iliaca block being used. This shows how frequently the use of the FIB impacts outcomes positively in the literature.

**Figure 3:**
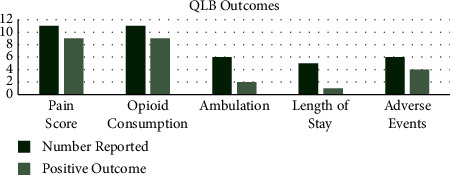
QLB outcomes. The chart displays the number of times an outcome was reported throughout the studies reviewed. The number of times a positive effect on the outcome was reported is plotted beside this to visualise the effects of the quadratus lumborum block being used. This shows how frequently the use of the QLB impacts outcomes positively in the literature.

Ambulation, length of stay, and adverse events were the next most commonly reported outcomes reported in 31%, 38%, and 52% of the studies, respectively. The number of times each factor was reported with how often a positive measure was recorded is visually represented in Figures 2 and 3 for FIB and QLB individually.

In the studies analysed, use of FIB resulted in pain score being positively impacted 83% of the time, opioid consumption 80%, ambulation 33%, length of stay 40%, and adverse events 63%. Ambulation was the only factor reported negatively when FIB was used, which was 33% of the time. In the studies analysed, use of QLB resulted in pain score being positively impacted 82% of the time, opioid consumption 82%, ambulation 33%, length of stay 20%, and adverse events 67%.

In the 4 papers which compared FIB and QLB against each other, all of the papers were positive for the use of fascial blocks; however, 3 of them could not determine which block was better overall. For the pain score outcome, 4 different scoring techniques were seen across the paper bank. Visual Analogue Scale (VAS), Numerical Rating Scale (NRS), Neuropathic Pain Diagnostic Questionnaire (DN4), and International Pain Outcomes (IPO), with variation among these also present in terms of timing postoperatively and at rest vs. on movement. Time until analgesia requested by patient was also utilised as a measurement of patient outcome within the studies. The use of various types of opioid was also noted across the literature (including morphine, oxycodone, and pethidine), with total opioid consumption, total cumulative opioid, and total morphine equivalent being recorded across the studies. Even the well-reported outcomes such as ambulation were recorded differently, for example, by the time to 1st stand, walking distance, 2-minute walking test, and time to mobilisation. In addition to the outcomes listed, there were an additional 8 other types of patient-reported outcomes utilised across the literature but with low frequency and, therefore, not significant enough to be analysed. These included outcomes such as sleep quality, sensory blockade, motor blockade, and range of motion.

Six variations in block dosage and two variations in block and surgical techniques for QLB were noted in the literature. In papers including the FIB, 12 different block dosages were observed, at least 4 different block techniques and 3 different THA techniques. The timings of the fascial blocks varied greatly across the studies analysed. In studies looking at FIB vs. control, 5 blocks were performed postoperatively, 5 were performed preoperatively, and 4 did not specify when they were performed. In studies investigating QLB vs. control, 2 blocks were performed postoperatively, 6 were performed preoperatively, and 3 did not specify when they were performed. In addition, 3 different types of primary anaesthesia were utilised. Some papers did not specify these parameters; hence, the description of “at least” is a set number of variations.

## 4. Discussion

It is clear that both the FIB and QLB have the ability to positively impact on a number of patient outcomes, particularly pain scores and opioid consumption which both influence rehabilitation and return to baseline function [10, 13–15]. It is difficult to confirm which block and with which parameters are optimal for patient outcomes due to the variable nature of the procedure. Looking at the FIB, 86% of the studies indicated positive outcomes compared to 83% of the QLB studies. In addition, when analysing the studies comparing QLB and FIB, 50% indicated that opioid consumption was reduced more when FIB was used over QLB and when QLB and FIB were used in conjunction over QLB alone. In the 4 studies comparing QLB and FIB, 1 out of 4 stated pain scores were significantly reduced in the FIB group [16]. However, the remaining outcomes were comparable [17–19]. From this, it could be argued that the FIB is more likely to reduce pain scores and opioid consumption in patients, the most commonly reported outcomes, which is deemed positive for overall recovery. Nevertheless, it is not a definitive argument. In terms of best dose, much variation is recorded and again this makes it difficult to definitely state the optimal dose. The most common dose with positive outcomes for FIB is 40 ml at 0.375% strength. For QLB, this is 20−30 ml at 0.25% strength. Dosages are analysed in more detail in pharmacological studies, which were not included in this review. With regards to timing of the block, in FIB studies, 36% completed the block preoperatively. Of these, 100% produced positive effects on outcomes. An additional 36% completed the block postoperatively with 60% of these studies produced positive effects. However, 28% did not specify when the dose was completed. Similarly, for QLB studies, 55% completed the block preoperatively, with 100% of these produced positive effects on outcomes. An additional 27% completed the block postoperatively with 66% producing positive effects on outcomes. As previously, 18% did not specify when the block was completed. From this, it could be said that preoperative fascial block administration is likely the most beneficial timing of administration. Bringing this together, FIB is likely the better block in terms of reducing pain scores and opioid consumption, with its optimal dose being 40 ml at 0.375%, administered preoperatively. Nevertheless, this narrative review cannot definitely confirm this due to its nature. However, this gives a basis for future work around these parameters. Furthermore, the blocks' effects on other outcomes such as ambulation and muscle strength are more ambiguous and may be hindering their widespread use [3, 20–22]. These outcomes also require more analysis.

### 4.1. Procedural Differences

There are a number of limitations prevalent in the literature which, therefore, impact this study. Within the studies reviewed, the country and centres at which the trials took place vary; therefore, some degrees of variation in terms of procedure may be expected. However, this could be holding back the standardisation of fascial blocks in THA. Reporting of the outcomes also varied between studies, both in terms of which outcomes were reported and how they were reported. This makes it difficult to collate mass results and produce clear, clinical guidelines since different reporting tools are used. Pain, for example, is reported frequently, using VAS, NRS, IPO, and DN4 questionnaire scoring systems. The timings postoperatively also differed with some studies recording at 6, 12, and 24 hours but some going as far as 48 hours. Scoring at rest and mobilisation also varied greatly between studies [16, 17]. Pain is a subjective measure and will vary with each individual no matter what type of reporting score is used, making it a difficult outcome to quantify and standardise the reporting. Therefore, to alleviate this as much as possible, a single, standardised pain score must be used when investigating fascial blocks to be able to quantify the analgesic benefits for THA. This paper is not designed to investigate optimal pain reporting systems, rather to highlight that it would be helpful to utilise the same approach across the board. Nevertheless, DN4 screening tool is specifically designed for neuropathic pain; therefore, it may not be optimal for postoperative pain [23]. The approach of VAS is relatively simple and easy to use with a 0–10 scale but may lack depth and miss relevant information [24]. NRS again uses scales, however, utilises 3 scales (0–10, 0–20, and 0–100) which may complicate a simple rating scale more than necessary [25]. Finally, the IPO system has been described as reliable and valid and has been widely used internationally for postoperative pain [26]. Despite this, VAS was reported most widely across the papers. To reduce reporting bias and validate results, a single pain reporting scale should be used.

Postoperative opioid consumption was also recorded in a variety of ways. Firstly, different opioids were used such as morphine, fentanyl, oxycodone, and pethidine [11, 27–29]. This may not influence results hugely and could be restricted by local guidelines and patient factors; however, for best practice and standardisation, utilising the same type of drugs across the board reduces the possibility of differences in results being due to reasons other than the fascial blocks [30]. Furthermore, the actual reporting of opioid consumption also varied. For example, some papers reported “total opioid consumption at 24 h,” “total morphine equivalent,” and “cumulative opioid consumption,” with the overall recorded time and total consumption also being reported differently throughout [18, 31]. Some articles reported the use of patient-controlled analgesia in addition to standard dosing [18]. Pain and opioid consumption were the most widely reported outcomes and are important factors to consider in the context of a new analgesic technique such as fascial blocks [4, 5, 7]. Nevertheless, further factors are evidently important such as ambulation, length of hospital stay, and patient satisfaction [14, 32, 33]. These were reported less consistently and again with variation. For example, ambulation distance, timed up and go test, and time to 1st stand and fall were all present across the literature and assess similar parameters but again the variability makes it difficult to quantify the effects of the fascial block on these particular factors [3, 10, 14]. In addition, secondary outcomes such as the sensory block and muscle strength were reported [31, 32], which may be of some importance; however, if ambulation and pain are more consistently reported to a more standardised degree, then there would be less need for their inclusion. If pain is low consistently, sensory blockade is evident and if ambulation is well-reported and positive, then muscle strength is likely good. This is to say, less outcomes, reported well and with reliability and validity, and is more beneficial than a range of variably reported outcomes.

Another source of variability is the surgical techniques utilised throughout the literature. The THA technique varied across papers, with some taking the classical posterolateral approach and others taking the anterior approach, which may be better for ambulation as the external rotators are not damaged [34]. This one difference could already invalidate an improved ambulation score as it is more difficult to determine whether that is due to the fascial block used or the surgical approach used. Moreover, the dose, timing, and technique of both the fascial blocks varied greatly between the papers. Dosages of both blocks varied from 20 ml to 40 ml volume and 0.2% to 0.375% strength. The timing of both blocks also varied with some being administered preoperatively and some postoperatively. The dosage, strength, and administration time of the blocks can influence how long they provide anaesthesia for and therefore impact the patient outcomes such as pain, opioid consumption, and ambulation.

### 4.2. Study Types

This review was designed to include as much literature as possible relevant to the topic of QLB and FIB for hip arthroplasty to highlight the current findings and to identify key areas for potential future work. Therefore, a variety of study types were included. This included retrospective, comparative, RCTs, and review papers. This may be a limitation of this study as the reliability of retrospective and comparative studies is questionable and review papers may complicate the results; nevertheless, the aim of this study was to provide as much literature as possible to portray the current stance in the field. The study of Metesky et al. was a retrospective study which involved looking at perioperative data for 39 patients undergoing THA [22]. They reviewed data from 20 patients who had a FIB and 19 who did not and concluded that FIB hindered ambulation. The retrospective nature of this study reduces its reliability as it is looking back on old notes and data and relying on good documentation and procedure, which the authors acknowledge. In addition, without knowing the exact methods carried out at the time of the procedures, it is difficult to standardise the results in this trial, which is evident from the limited methods and results' section. In future, studies like this could be removed from the review of the literature, as due to their poor reliability, they may be responsible for increasing the negative scores of the FIB unnecessarily. On the contrary, Wang et al. was a double-blinded RCT comparing FIB and QLB which is more reliable in its results. This is due to a detailed methods' section, which clearly defines how the groups were created, why THA approach was utilised, which anaesthesia, and the dose and timings of the block [19]. With a sample size of 100 and the outcome assessor and surgeons blinded to the blocks utilised, this paper is much more reliable and contributes more to this reviews' results. It may have been more beneficial to include solely RCT's for this paper; however, the narrative approach here is aimed at giving as much of an overview as possible as a platform for readers and future work. From the 29 papers in this review, the majority are considered good quality, with 52% being RCT. Retrospective and review articles made up of 21% of the studies each and comparative and case studies 3% each. This displays that the results are coming from a range of studies, which may limit the reliability; however, with such strong results in favour of the blocks, the narrative stance of this article is achieved, giving a good overview of the current stance in the field.

### 4.3. Future Work/Proposal

From the literature, it is clear that these fascial blocks have potential in improving patient outcomes. The impact of both the FIB and QLB on patient outcomes is largely positive, meaning reported pain scores and postoperative opioid consumption have been reduced most of the time, aiding better rehabilitation and recovery following THA. As described in the methods, a positive effect means an outcome has been impacted in a way which is deemed good for the patient's recovery. Reduction in pain scores and opioid consumption is deemed positive as it is likely to improve patient mobilisation and rehabilitation, which are good prognostic factors in THA [4–6]. Other outcomes that were reported included ambulation, length of stay, and adverse events. These were less commonly reported throughout the literature, and the effects of the blocks on them displayed more variation than pain and opioid consumption. Nevertheless, positive effects were seen in all of these outcomes, as can be visualised in Figures 2 and 3. However, more detailed work is required in these areas to give a broader idea of the effects of the fascial blocks. This would be a basis for future work and would be easier with an established timing and dosage of the respective blocks. The optimal technique and dosage is not absolutely clear from this review; however, more consistent positive effects were seen with the FIB, administered preoperatively at a dose of 40 ml 0.375%. Future work requires a large, multicentre, randomised control trial which aims to standardise the optimal technique for FIB and QLB. This may be difficult to achieve due to the number of variables including dose, timing, block technique, and surgical technique. Nevertheless, finding the best combination would be advantageous in improving patient recovery after THA [9, 14, 16]. A large trial where the techniques are standardised is required to reduce the chance of confounding variables being the reason for the positive results over the fascial blocks.

## 5. Conclusions

It is clear how difficult managing postoperative pain following a THA can be and it has a significant impact on patient rehabilitation [2, 3]. Finding the optimal analgesic regime and, therefore, improving function thereafter is extremely important for the patient [2, 3, 5]. It is clear that fascial blocks have a positive effect on recovery, mainly due to reduced pain and opioid consumption [15, 20, 21]. Finding the optimal analgesic block regime and standardising a protocol for its use would be beneficial in maximising the potential of these techniques. Larger multicentre trials will be required with less variation between techniques of the blocks and the surgery to identify the most effective way to utilise the fascial block and improve patient rehabilitation and outcomes following THA.

## Figures and Tables

**Table 1:** Eligibility criteria for studies.

Eligibility criteria	Justification
English language only	Avoidance of translation issues
Past 5 years only	Most up-to-date and accurate information
Total hip arthroplasty only	Very common surgery and is the focus of the review
Randomised control trials	Produce reliable results with limited bias
Case studies included	QLB in early use with THA may provide valuable information in conjunction with other literature
Review articles included	Increases information base for narrative of the literature
Knee arthroplasty or other surgery excluded	Not the focus of the review and reduces specificity
Paediatrics excluded	Different cohort and reduces specificity

The table displays the criteria used to exclude/include studies for this review. The right column explains the justification for the criteria.

**Table 2:** Summary of study findings.

Paper	Fascial block	Effect of block on patient outcomes	Overall effect of block
Abduallah 2020	QLB	↓↓Pain score	+
		↓↓Opioid consumption	

Brixel 2021	QLB	↓Pain score	±
		↓Opioid consumption	

Kim 2021	QLB	↓↓Pain scores	+

Wang 2022	QLB	↓↓Pain scores	+
		↓↓Opioid consumption	

Hussain 2022	QLB	↓Pain scores	±
		↓Opioid consumption	

He 2020	QLB	↓↓Pain scores	+
		↓↓Opioid consumption	

Huda 2022	QLB	↓↓Pain scores	+
		↓↓Opioid consumption	

Kukreja 2019	QLB	↓↓Pain scores	+
		↓↓Opioid consumption	

Kukreja 2019	QLB	↓↓Pain scores	+
		↓↓Opioid consumption	

Ramprasad 2020	QLB	↓↓Pain scores	+
		↓↓Opioid consumption	

Hu 2022	QLB	↓↓Pain scores	+
		↓↓Opioid consumption	

Bober 2020	FIB	↓↓Pain scores	±
		↓Opioid consumption	

Carella 2022	FIB	↓↓Pain scores	+
		↓↓Opioid consumption	

Gola 2021	FIB	↓↓Pain scores	+
		↓↓Opioid consumption	

Badawai 2022	FIB	↓↓Pain scores	+
		↓↓Opioid consumption	

Khan 2021	FIB	↓↓Pain scores	+

Zheng 2021	FIB	≅Pain scores	+
		↓Opioid consumption	

Cai 2019	FIB	↓↓Pain scores	+
		↓Opioid consumption	

Gao 2019	FIB	↓↓Pain scores	+
		↓↓Opioid consumption	

Zhang 2019	FIB	↓↓Pain scores	+
		↓↓Opioid consumption	

Wang 2021	FIB	↓↓Opioid consumption	+

Sabra 2019	FIB	↓↓Pain scores	+
		↓↓Opioid consumption	

Liu 2020	FIB	↓↓Pain scores	+
		↓↓Opioid consumption	

Metesky 2019	FIB	↓↓Ambulation	−

Li 2022	FIB	↓↓Pain scores	+

Wang 2022	FIB vs. QLB	↓↓Pain scores FIB ≅ QLB	+
		↓↓Opioid consumption FIB ≅ QLB	

Nassar 2021	FIB vs. QLB	↓↓Pain scores FIB ≅ QLB	+
		↓↓Opioid consumption FIB > QLB	

Xia 2021	FIB + QLB vs. QLB	↓↓Pain scores FIB + QLB > QLB	+
		↓↓Opioid consumption FIB + QLB > QLB	

Hashmi 2022	FIB vs. QLB	↓↓Pain scores FIB ≅ QLB	+
		↓↓Opioid consumption FIB ≅ QLB	

The table displays the effects of the blocks on the core outcomes of each paper. This table displays all the studies used in this review, what block type they were reporting, the effects on the core outcomes, and the overall stance of the paper. Pain scores and opioid consumptions are the most widely reported outcomes and have, therefore, been included in this table. One exception is the study by Metesky et al., 2019, which focused solely on ambulation. Positive stance requires reduction in one outcome such as pain score or opioid consumption, neutral means no significant findings, and negative requires a change in an outcome which worsens postoperative outcomes. A negative example in this table is a reduction in ambulation. (*Notes*. ↑↑/↓↓ = significant increased/reduction, ↑/↓ = increase/reduction (not significant), + = positive, − = negative, ± = neutral, ≅ = comparable, and > = better than).

## Data Availability

The data within this article has been created from the results of the articles reviewed. These are all included in the reference section of this article.
